# Draft Genome Sequences for Three *Ophiostoma* Species Acquired during Revisions of Australian Plant Pathogen Reference Collections

**DOI:** 10.1128/mra.00175-22

**Published:** 2022-05-12

**Authors:** Conrad Trollip, Angus J. Carnegie, Brendan Rodoni, Jacqueline Edwards

**Affiliations:** a School of Applied Systems Biology, La Trobe University, Bundoora, Victoria, Australia; b Agriculture Victoria, Department of Jobs, Precincts, and Regions, AgriBio Centre, Bundoora, Victoria, Australia; c Forest Science, NSW Department of Primary Industries-Forestry, Parramatta, New South Wales, Australia; Vanderbilt University

## Abstract

The fungal genus *Ophiostoma* contains numerous species that share close associations with wood-boring insects, a relationship with important consequences for global biosecurity. Here, we provide draft genomes for three *Ophiostoma* species within the well-known Ophiostoma ulmi complex. These resources are valuable for future research efforts related to *Ophiostoma* and the establishment of biosecurity-focused databases.

## ANNOUNCEMENT

The genus *Ophiostoma* (order *Ophiostomatales*, class *Sordariomycetes*, phylum *Ascomycota*) contains approximately 134 species (1) and is renowned for harboring economically important fungi, the most notable of which are the Dutch elm disease pathogens Ophiostoma ulmi and Ophiostoma novo-ulmi (2). *Ophiostoma* species are vectored by bark- and wood-boring insects, a relationship that is hypothesized to be strongly coevolved and host specific (3). The potential for global range expansions into novel ecosystems, caused by insect-mediated dispersal combined with increased human trade (4), has resulted in the regular inclusion of *Ophiostoma* species (and/or their vectors) on high-priority pest lists by biosecurity agencies (e.g., see references 5 and 6). To establish a biosecurity-focused database for Australia, we recently began the revision of ophiostomatalean specimens lodged in Australian plant pathogen reference collections (7). Here, two specimens (DAR52683 and DAR52684) were obtained from the New South Wales (NSW) Plant Pathology and Mycology Herbarium, both originally lodged as Ophiostoma piceae. These specimens were sampled from Lophozonia cunninghamii (= Nothofagus cunninghamii) collected in the Arve Valley in Tasmania, Australia, in 1985 (collected by G. Kile). An additional specimen (VPRI43877) obtained from the Victorian Plant Pathology Herbarium was originally lodged as a *Sporothrix* sp. collected from Eucalyptus globulus in Victoria, Australia, in July 2020 (collected by D. Smith).

DNA extraction, library preparation, and sequencing were performed in house and followed the methods described by Trollip et al. (7). Briefly, preserved specimens were successfully revived before 7-day-old cultures were used to inoculate 40-mL potato dextrose broth (Oxoid, UK). Liquid cultures were grown on a shaking incubator at room temperature (150 rpm) for 72 h before mycelia were harvested and freeze-dried for DNA extractions using the Promega Wizard genomic DNA purification kit (Promega, USA) with the protocol for isolation from plant tissue. Libraries with an average insert size of 300 bp were prepared with the NextFlex Rapid XP DNA-sequencing (DNA-Seq) kit (Perkin Elmer, USA) and sequenced in paired-end format (2 × 150 bp) on a NovaSeq 6000 system (Illumina, USA). Raw sequencing reads were quality checked and trimmed using FastP (8), followed by *de novo* genome assembly with read error correction and *k*-mer values of 33, 55, 77, 97, and 111 specified in SPAdes v3.14.1 (9). Finally, assembled genomes were used to extract commonly sequenced barcodes (namely, internal transcribed spacer [ITS] and translation elongation factor 1-α [TEF1-α]) for species identification by mapping *Ophiostomatales* reference sequences using BBMap (10), as described by Trollip et al. (7).

**FIG 1:**
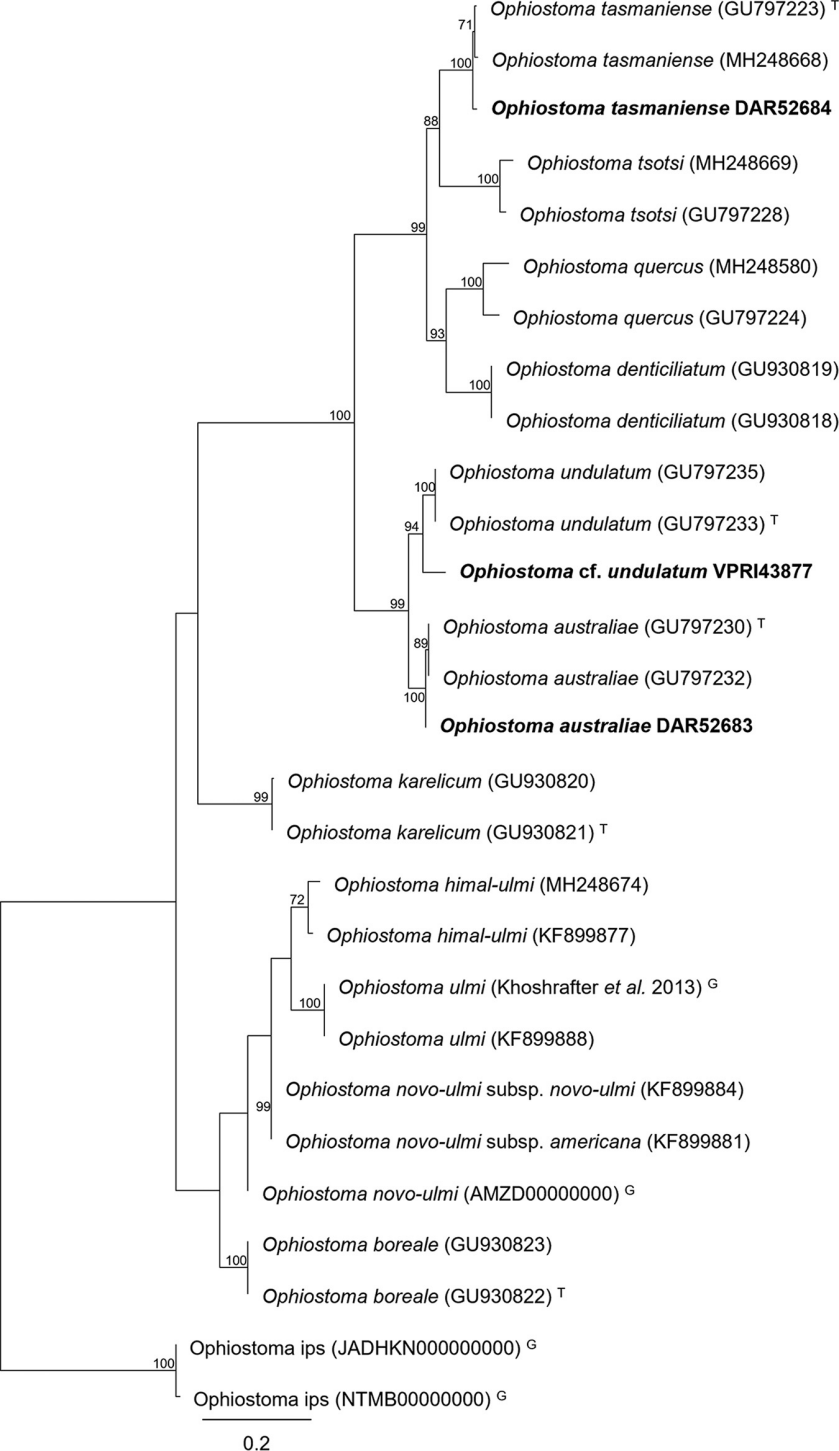
Maximum likelihood phylogeny of TEF1-α used to identify the sequenced *Ophiostoma* strains. Multiple sequence alignment was performed using MAFFT v 7 (14) before substitution model selection was performed with ATGC:SMS online (15). Maximum likelihood phylogenetic inference was performed with RaxML v8.2.11 (16) using the GTR+gamma model. Mapping, alignment, and phylogenetic inference were all conducted within Geneious Prime v2022.0.2 (Biomatters Ltd.) using the aforementioned software as plug-ins. Strains sequenced in this study are highlighted with bold text. T, ex-type isolate sequence; G, sequence extracted from publicly available genome data (7, 17–19).

Here, we present the draft genome sequences of three *Ophiostoma* species, namely, Ophiostoma australiae DAR52683, Ophiostoma tasmaniense DAR52684, and Ophiostoma cf. undulatum VPRI43877 (Fig. 1), all of which are known from Australia and reside in the O. ulmi complex (11, 12). Genome quality and assembly statistics were evaluated using QUAST v5.0.2 (13) and are summarized in Table 1. The benchmarking universal single-copy orthologs (BUSCO) results (with the sordariomycetes_odb10 data set) showed the genomes being 96.9% (DAR52684), 97.0% (DAR52683), and 97.2% (VPRI43877) complete, while gene prediction (–fungus) reported very similar numbers of predicted genes for O. australiae and O. cf. undulatum (8,565 and 8,560 predicted genes, respectively), with O. tasmaniense having a slightly lower number of 8,454. The genomic data for these three species, which are currently considered nonpathogenic (11), represent an important addition to *Ophiostomatales*-focused resources because of the close phylogenetic association with the O. ulmi and O. novo-ulmi pathogens. The O. australiae, O. tasmaniense, and O. cf. undulatum genomes should prove to be a valuable resource for future comparative studies within the O. ulmi complex.

**TABLE 1:** Genome summary statistics for scaffolds of more than 500 bp analyzed with QUAST v5.0.2 (12)

Taxon	Strain	GenBank accession no.	No. of reads after quality control	No. of scaffolds	Estimated size (Mb)	*N*_50_ (bp)	GC content (%)	Coverage (×)
O. australiae	DAR52683	JAKRGJ010000000	42,003,190	34	31.42	1,938,765	53.1	200
O. tasmaniense	DAR52684	JAKRGK010000000	32,802,716	45	32.57	1,470,959	50.8	151
O. cf. undulatum	VPRI43877	JAKRGL010000000	21,359,904	57	31.52	1,120,264	52.2	101

### Data availability.

Quality-trimmed sequence reads (SRA accession numbers SRR18010332, SRR18010333, and SRR18010334) and draft genomes have been deposited in DDBJ/EMBL/GenBank under BioProject accession number PRJNA805285. The accession numbers for each genome are presented in Table 1. Partial TEF1-α sequences used for phylogenetic analysis are also available (GenBank accession numbers ON101404 to ON101406).
